# Tubulointerstitial nephritis antigen-like 1 protein is downregulated in the placenta of pre-eclamptic women

**DOI:** 10.1186/s12014-017-9144-2

**Published:** 2017-03-21

**Authors:** Sheon Mary, Mahesh J. Kulkarni, Savita S. Mehendale, Sadhana R. Joshi, Ashok P. Giri

**Affiliations:** 1 Division of Biochemical Sciences, CSIR-National Chemical Laboratory, Pune, Maharashtra 411008 India; 2 Department of Gynecology, Bharati Vidyapeeth Medical College, Pune, Maharashtra 411043 India; 3 Department of Nutritional Medicine, Interactive Research School for Health Affairs, Bharati Vidyapeeth University, Pune, Maharashtra 411043 India

**Keywords:** Pre-eclampsia, Tubulointerstitial nephritis antigen-like 1 protein, Human placenta, Matricellular protein, Proteomics

## Abstract

**Background:**

Tubulointerstitial nephritis antigen-like 1 protein (TINAGL1), is a matricellular protein, known to play role in cell adhesion and cell receptor interaction. Research related to TINAGL1 is limited to cell culture and animal models. Demonstration of TINAGL1 as a positive regulator of angiogenesis and its expression in the decidua of postimplantation mouse uterus, prompted us to validate its expression in human placenta during impaired angiogenesis in pre-eclamptic condition.

**Methods:**

Placental tissue from normotensive (n = 25) and pre-eclamptic (n = 25) pregnancies were used to study the differentially expressed proteins by two-dimensional gel electrophoresis and TINAGL1 protein was validated with Western blotting.

**Results:**

A total of 55 protein spots were differentially expressed (fold change >1.5, p < 0.05), of which 27 were upregulated and 28 were downregulated in the pre-eclamptic placenta. TINAGL1 was found to be downregulated in pre-eclamptic compared to normotensive pregnant women.

**Conclusion:**

This is the first study reporting TINAGL1 to be present in human placenta and differentially expressed in pre-eclamptic condition. The functional role of TINAGL1 in association to human pregnancy needs to be explored further.

**Electronic supplementary material:**

The online version of this article (doi:10.1186/s12014-017-9144-2) contains supplementary material, which is available to authorized users.

## Background

Tubulointerstitial nephritis antigen-like 1 protein (TINAGL1) also known as Lipocalin 7 (LCN7) or adrenocortical zonation factor-1 (AZ1) or tubulointerstitial nephritis antigen related protein (TIN-ag-RP) is a matricellular protein. TINAGL1 is similar to tubulointerstitial nephritis antigen (TINAGL) in its protein domain composition, i.e. two EGF-like domains and a proteolytically inactive cathepsin B-like domain [1]. Unlike TINGAL, its expression is not limited to kidney, but found in a variety of organs, such as vascular smooth muscle [1], glomerular basement membrane [1], uterine capillaries [2], adrenocortical cells [3] and lung capillary endothelium [4]. As a matricellular protein, it interacts with other structural matrix proteins such as laminin, collagen, and fibronectin and plays a role of a ligand for cell integrins receptors such as α_1_β_1_, α_2_β_1_ and α_5_β_1_ [3]. Brown et al. [5], using murine endothelial cell lines and zebrafish embryo demonstrated that TINGAL1 is a positive regulator of angiogenesis that increases endothelial cell invasion, angiogenic sprouting and sensitivity of TGF-β. The demonstration of marked expression of TINGAL1 in the decidua of postimplantation mouse uterus [2] and the blastocoelic surface of trophectoderm [6] has instigated the possible role of this protein in pregnancy.

Pre-eclampsia (PE) complicates 5–8% pregnancies and is a leading cause of maternal and perinatal morbidity and mortality worldwide. PE is diagnosed by the new onset of hypertension (>140/90 mmHg) accompanied with new onset of proteinuria (>300 mg/24 h) after 20 weeks of gestation, or in the absence of proteinuria, hypertension accompanied with new onset of any of the following symptoms viz. thrombocytopenia, renal insufficiency, impaired liver function, pulmonary edema and visual or cerebral symptoms [7]. Although the origin of PE remains enigmatic, the primary underlying cause is the abnormal placentation due to inefficient trophoblast invasion into the spiral artery. The reduced spiral artery remodeling leads to decreased blood flow to the placenta, making it more hypoxic. As a consequence of oxidative stress, inflammation and apoptosis varying pathophysiological problems such as immunological, generalized endothelial dysfunctions and placental ischemia arise in these women [8, 9]. The role of the placenta in pre-eclampsia is undeniable, thus needs to be explored further. We investigated the placental proteome of pre-eclamptic women using proteomic techniques and found expression of TINAGL1 in the placenta.

## Methods

### Sample collection

Patients recruited for the study were classified as PE if they showed systolic and diastolic blood pressures >140 and 90 mmHg, respectively, with the presence of proteinuria (>1+ or 300 mg/24 h) on a dipstick test and was confirmed by repeated recording of the blood pressure with an interval of 6 h. Women with an indication of chronic hypertension, type 1 or 2 diabetes mellitus, renal or liver diseases and seizure disorder were excluded from the study. The sample set used in this study is same as our earlier study [10]. All samples were collected from Department of Obstetrics and Gynecology, Bharati Hospital, Pune, Maharashtra, India with informed consent from the patient and were approved by the Bharati Vidyapeeth Medical College Institutional Ethical Committee. Small sections of placental tissues from normotensive and pre-eclamptic pregnancies were collected immediately after delivery and rinsed with phosphate buffered saline to wash off the blood, snap frozen in liquid nitrogen and stored at −80 °C until further use.

### Protein extraction, separation, and identification

Frozen placental tissue was pulverized, and dissolved in lysis buffer (100 mg/0.5 ml) (7 M urea, 2 M thiourea, 4% 3-[(3-cholamidopropyl) dimethylammonio]-1-propanesulfonate, 2% Dithiothreitol). The homogenate was centrifuged and the supernatant used as a source of placental proteins. Two technical replicates of three pools of normotensive (n = 18) were compared with three pools of PE (n = 18) protein samples using two-dimensional electrophoresis (2DE). For the first dimension, immobilized pH gradient (IPG) strips 4–7 and 12% Tris–Glycine SDS-PAGE gels for the second dimension electrophoresis were used. The stained gels images were scanned on GS-800 Densitometer and analyzed for spot intensity data by PDQuest software (Bio-Rad Laboratories, Hercules, CA, USA). Protein spots excised from 2DE were de-stained, followed by reduction and alkylation. Digested peptides were analyzed in LC-MS^E^ using a NanoAcquity ultra-performance liquid chromatography (UPLC) system coupled to an SYNAPT high definition mass spectrometer (Waters, Milford, MA, USA). Nano C18 reversed phase column (1.7 μm particle size, i.d. 75 μm and length 250 mm) (Waters) and binary solvent system (99.9% water and 0.1% formic acid (mobile phase A) and 99.9% acetonitrile and 0.1% formic acid (mobile phase B)) was used for peptide separation. Data were processed and searched using ProteinLynx Global Server 2.4 (PLGS) software (Waters). UniProtKB *H. sapiens* proteome (UP000005640), including only reviewed sequences (47,869) from UniprotKB/SwissProt (updated version of *H. sapiens* proteome, 2008) was used to search against the MS data. A fixed modification included carbamidomethyl C and variable modifications such as oxidation M, deamidation N, and deamidation Q. Automatic setting of PLGS was used for mass accuracy of precursor and fragment ions. Identification was done with minimum two peptides and FDR of 1%. For each protein identified, a number of unique peptides for a given protein was calculated using PepServe bioinformatics tool. The dataset (PASS00711) was uploaded to publicly accessible PeptideAtlas database (www.peptideatlas.org, Institute for Systems Biology, Seattle, WA).

### Western blot analysis

For validation of TINAGL1, placental proteins from normotensive (n = 25) and pre-eclamptic (n = 25) were separated on SDS-PAGE and blotted onto one single PVDF membrane (Millipore Corporation, Billerica, MA, USA). Multi-strip Western blotting protocol was performed all 50 individual protein samples as mentioned in Aksamitiene et al. [11]. All antibodies were purchased from Santa Cruz Biotechnology (Santa Cruz, CA, USA) and used as follows: primary antibody for tubulointerstitial nephritis antigen-like 1 protein (1:2000), beta-actin (1:5000) as housekeeping, secondary anti-goat conjugated to biotin (1:10,000) and streptavidin conjugated HRP (1:20,000). Detection was performed by chemiluminescent ECL reagent (GE Healthcare, Little Chalfont, BUX, UK) and signals were detected with CCD imaging system (Syngene, Cambridge, UK) camera. Analysis of total signal intensity of Western blot bands was performed with Image Studio Lite version 4.0 (Li-Cor Biosciences, Lincoln, NE, USA) using local background subtractions.

### Statistical analysis

Analysis of statistical significance of differential expressed protein 2D protein spots and Western blot protein bands were carried out using one-tailed Student’s t test. Analyzes were performed using SPSS/Pc package software version 18.0 (Chicago, IL, USA) and results were considered statistically significant if *p* ≤ 0.05.

## Result

In the typical 2DE gels over 228 protein spots were detected in placental protein extracts, of which 55 protein spots were differentially expressed. We identified 27-upregulated and 28-downregulated protein spots in PE compared to the normotensive placenta. It was notable that 9-protein spots were upregulated more than twofold in PE, while 7-protein spots were upregulated more than twofold in normotensive placental tissue with high confidence (*p* ≤ 0.05). The identified differentially expressed placental protein spots in pre-eclamptic and normotensive proteins with their molecular weight and isoelectric point, fold change with *p* value are listed in Additional file 1: Figure S1. Differentially expressed proteins showed increased expression of molecular chaperones such as 60 kDa HSP mitochondrial, heat shock cognate 71 kDa protein, HSP70 kDa, while of HSP90, endoplasmin and hypoxia up-regulated protein-1 showed decreased expression in PE. The endoplasmic reticulum stress related proteins such as endoplasmin, hypoxia up-regulated protein-1, transitional endoplasmic reticulum ATPase were downregulated, and protein disulphide isomerase A6 and thioredoxin domain containing protein 5 were upregulated in PE group. Alpha-actinin family of proteins, alpha-actinin-1, -2, -3 and -4 were observed to be downregulated in pre-eclamptic group. These proteins are actin-binding proteins and have various functions in different cell types such as platelet activation and degranulation, blood coagulation, hypoxia and apoptosis.

**Fig. 1:**
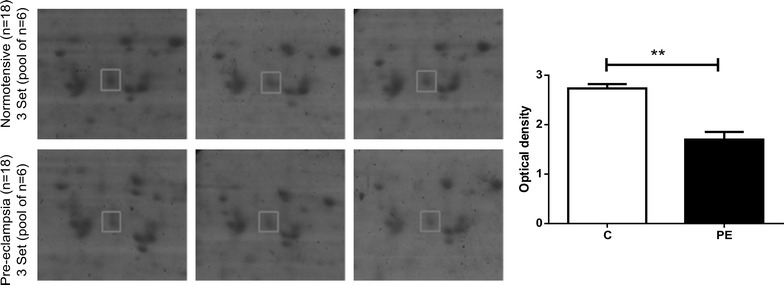
Magnified images of differentially expressed TINAGL1 spots. The *bar chart* represents mean ± SE of the optical density of the spot for the three sets of normotensive (C) and pre-eclamptic (PE). **p < 0.01

**Fig. 2:**
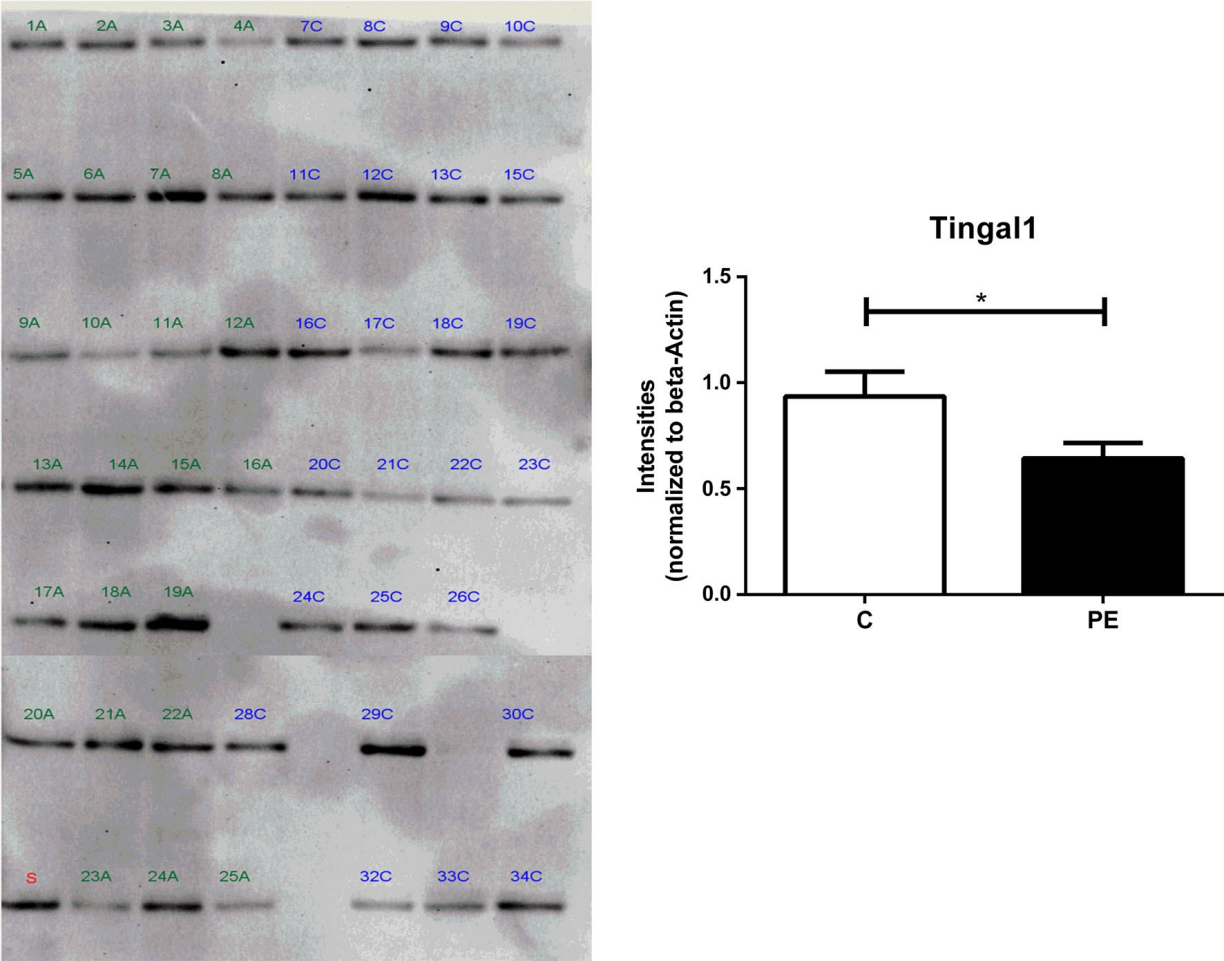
Western blot for tubulointerstitial nephritis antigen-like 1 protein. In the image, PE samples are marked with A (*1*–*25A*) and normotensive with C (*7*–*34C*), and standard sample (S) made by pooling all the individual sample. The *graph* represents intensities of normotensive (C) and pre-eclamptic pregnancies (PE) normalized to beta-actin. The *bar* represents mean ± SE of 25 each individual of the normotensive and pre-eclamptic placenta (*p < 0.05). Additional file 2: Table S1 shows the calculation for beta-actin normalization

This is the first study reporting the expression of TINAGL1 in pre-eclamptic condition. The conventional protein separation on two-dimensional SDS-PAGE, identified TINAGL1 protein spot with 6.5 isoelectric point and molecular weight of approximately 52 kDa. It was down-regulated in the placenta of pre-eclamptic women with a fold change of 1.6 (*p* = 0.004) (Fig. 1). Mass spectrometric analysis identified 68 and 54 peptides with a sequence coverage of 61 and 42% in normotensive and pre-eclamptic patients, respectively (Additional file 2: Table S1). Validation of TINGAL1 was performed by western blot of individual normotensive (n = 25) and pre-eclamptic (n = 25) patient’s placenta. Similar to 2DE results, we observed 1.5 (*p* = 0.022) fold decrease of TINGAL1 in the placenta of pre-eclamptic women in Western blot analysis (Fig. 2; Additional file 3: Table S2).

## Discussion

TINAGL1 is reported to be present in other tissue as well as in placenta [12]. Human protein atlas shows its maximum RNA expression in placenta. TINAGL1’s molecular functions are not well explored in human pregnancy or pre-eclampsia. Most of its biological role is studied in the mouse. Human TINAGL1 shows 90% amino acid sequence identity with mouse protein. TINAGL1 acts as a ligand for integrins α1β1, α2β1 and α5β1, suggesting its role in cell adhesion [2, 3]. Expressions of these integrins are important for trophoblast invasion [13]. Recently, *Tinagl1*−*/*− mice were shown to have impaired fertility during pregnancy [14].

Angiogenesis is one of the major events during pregnancy. Imbalance in angiogenic factors is one of the major events in the pathogenesis of pre-eclampsia [15]. The discovery of TINAGL1 as a pro-angiogenic factor in in vitro and in vivo models of angiogenesis [5], has set off its possibility to play a role in angiogenesis during pregnancy. Low level of TINAGL1 in the placenta of pre-eclamptic women might be one of the factors in impaired angiogenesis and cell adhesion which needs to me explored further. Studying its molecular mechanism in earlier stages of gestation in the human will be challenging and one needs to rely on animal models of pre-eclampsia.

## Conclusion

In conclusion, during our preliminary investigation on the placental proteome of pre-eclamptic and normotensive women by two-dimensional gel electrophoresis, we identified tubulointerstitial nephritis antigen-like 1 protein as one of the differentially expressed proteins that are hitherto not studied in pre-eclampsia. Hence, we validated the expression of this protein in placental tissue (n = 50) of pre-eclamptic and normotensive subjects.

## Additional files

**Additional file 1: Figure S1.** Representative 2D SDS-PAGE gel of pre-eclamptic and normotensive Coomassie blue stained gels and protein spot numbered on the images. LC-MS^E^ identification of differentially expressed proteins in normal versus pre-eclamptic pregnancies.

**Additional file 2: Table S1.** PLGS protein identification data including MW, pI, the number of peptides, PLGS score, coverage, modifications, MS/MS fragmentation, and sequence of peptides.

**Additional file 3: Table S2.** Western Blot normalization calculations.

## Abbreviations

PE: pre-eclampsia
TINAGL1: tubulointerstitial nephritis antigen-like 1 protein
2DE: two-dimensional electrophoresis
