# Supplementary material for: Tubulointerstitial nephritis antigen-like 1 protein is downregulated in the placenta of pre-eclamptic women
Source: Clin Proteomics. 2017 Mar 21;14:8. doi: 10.1186/s12014-017-9144-2 (PMC5361709; doi:10.1186/s12014-017-9144-2)
Supplement: Supplementary file 1 — Additional file 1: Figure S1. Representative 2D SDS-PAGE gel of pre-eclamptic and normotensive Coomassie blue stained gels and protein spot numbered on the images. LC-MSE identification of differentially expressed proteins in normal versus pre-eclamptic pregnancies. [file 12014_2017_9144_MOESM1_ESM.pdf]

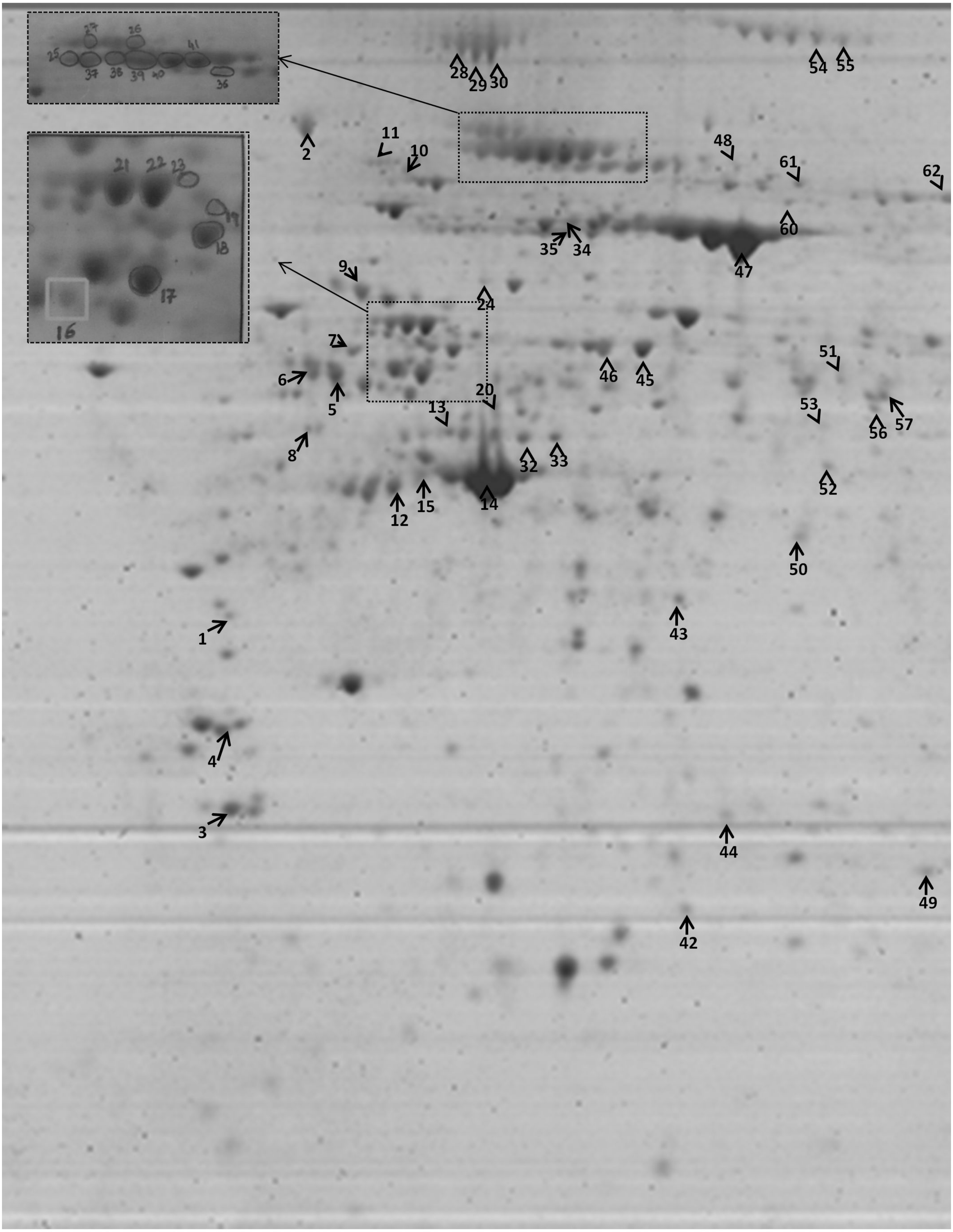

27
26
25
41
37
38
39
40
36
21
22
23
19
18
17
16
28
29
30
54
55
2
11
10
48
61
62
60
35
34
47
9
24
7
6
5
46
45
51
20
13
53
57
56
8
32
33
52
15
14
12
50
1
43
4
3
44
49
42

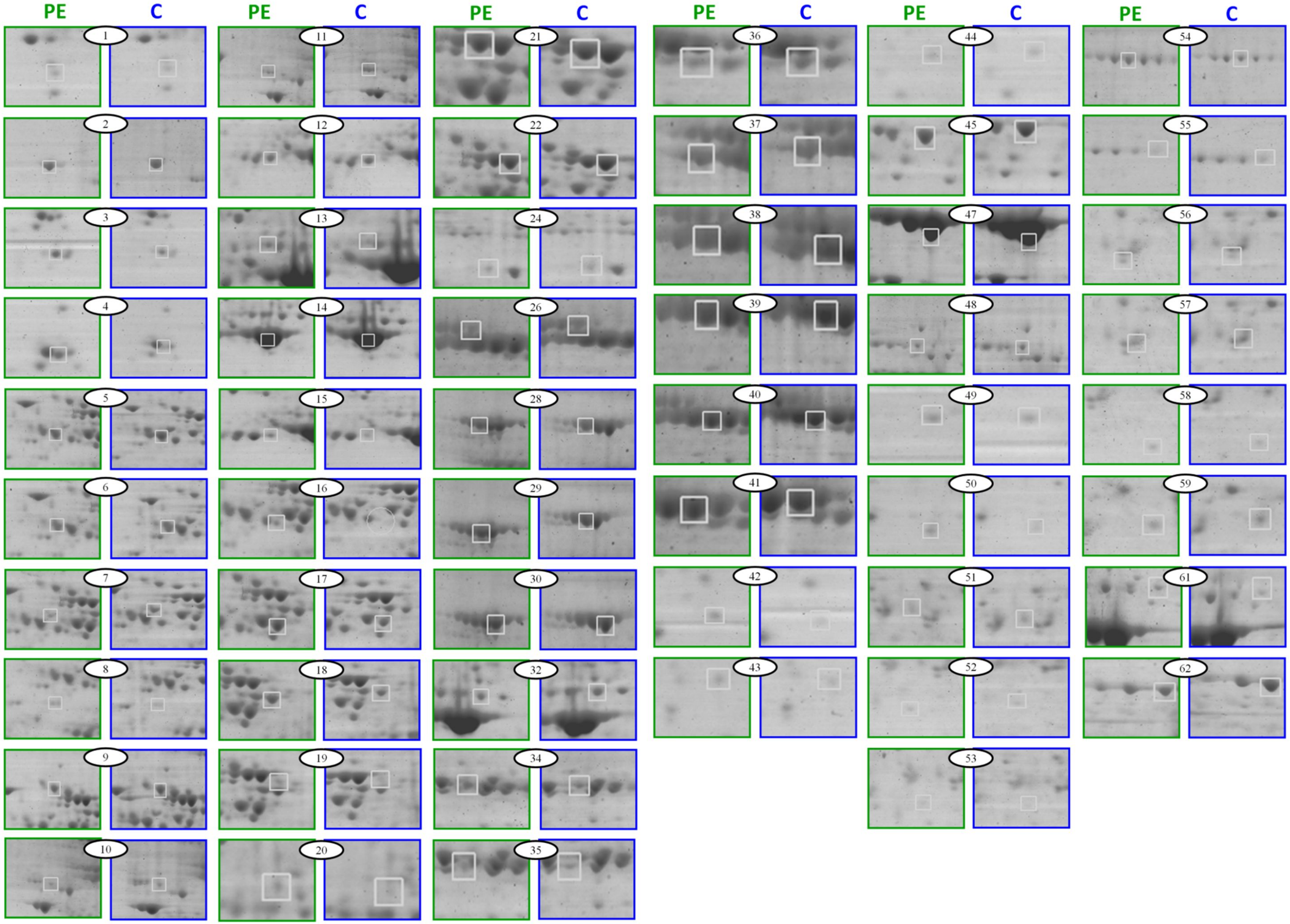
PE
C
PE
C
PE
C
PE
C
PE
C
PE
C
1
2
3
4
5
6
7
8
9
10
11
12
13
14
15
16
17
18
19
20
21
22
24
26
28
29
30
32
34
35
36
37
38
39
40
41
42
43
44
45
47
48
49
50
51
52
53
54
55
56
57
58
59
61
62

Table: LC-MS^E identification of differentially expressed proteins in normal versus pre-eclamptic pregnancies. Protein identification was done using PLGS and the number of unique peptides identified using PepServe software. In the column with fold change, ↑ represents up regulation and ↓ represents down regulation. () consists of total peptide identified by PLGS which includes more than twice identification of same peptide.

Spot No.	Accession No.	Protein description	MW	pI	Sequence coverage (%)	Unique peptide (Total peptide identified)	Fold change PE/C	*p* value
**1**	P09493	Isoform 3,9,8 of Tropomyosin alpha 1 chain	32855	4.5	33.1	(17)	1.5↑	0.031
**2**	P14625	Endoplasmin	92411	4.6	63.6	22(198)	1.8↓	0.007
**3**	P63104	14 3 3 protein zeta delta	27727	4.5	64.1	6(46)	1.6↑	0.049
	Q04917	14 3 3 protein eta	28201	4.6	28.5	3(15)		
	P61981	14 3 3 protein gamma	28284	4.6	23.9	2(15)		
**4**	P67936	Tropomyosin alpha 4 chain	28504	4.5	22.2	3(8)	2.5↑	0.289
**5**	P01009	Alpha 1 antitrypsin	46707	5.2	59.6	12(103)	1.7↓	0.013
**6**	P01009	Alpha 1 antitrypsin	46707	5.2	54.8	15(84)	1.5↓	0.000
**7**	P07437	Tubulin beta chain	49638	4.6	73.9	4(55)	1.9↓	0.000
**9**	P01009	Alpha 1 antitrypsin	46707	5.2	65.1	16(91)	1.5↓	0.287
**10**	P21980	Protein glutamine gamma glutamyltransferase 2	77279	4.9	33.9	11(25)	2.1↓	0.004
**11**	P08238	Heat shock protein HSP 90 beta	83212	4.8	58.1	8(96)	1.7↓	0.005
	P07900	Heat shock protein HSP 90 alpha	84606	4.7	52.9	7(80)		
**12**	P60709	Actin cytoplasmic 1	41709	5.1	69.3	(42)	2.8↓	0.015
**13**	P63261	Actin cytoplasmic 2	41765	5.2	22.4	(10)	2.7↑	0.021
**14**	P60709	Actin cytoplasmic 1	41709	5.1	80.8	(115)	3.0↓	0.156
	P63261	Actin cytoplasmic 2	41765	5.2	80.8	(155)		
**15**	P60709	Actin cytoplasmic 1	41709	5.1	72.8	(52)	1.8↑	0.313
	P68032	Actin alpha cardiac muscle 1	41991	5.1	65.8	(50)		
**16**	Q15084	Protein disulfide isomerase A6	48091	4.8	29.8	8(10)	3.1↑	0.000

**17**	Q15084	Protein disulfide isomerase A6	48091	4.8	48.6	8(16)		
**18**	P17661	Desmin	53503	5.0	61.1	16(49)		
	P02774	Vitamin D binding protein	52929	5.2	61.6	8(48)		
	P17661	Desmin	53503	5.0	58.1	9(51)		
**20**	Q8NBS9	Thioredoxin domain containing protein 5	47598	5.6	45.8	10(38)	3.1↑	0.003
**24**	P10809	60 kDa heat shock protein mitochondrial	61016	5.6	75.9	22(74)	1.6↑	0.358
**26**	O43707	Alpha actinin 4	104788	5.1	81.6	25(112)	3.2↓	0.041
**28**	P12109	Collagen alpha 1 VI chain	108461	5.1	61.7	27(138)	1.5↓	0.001
	Q05707-3	Isoform 3 of Collagen alpha 1 XIV chain	183037	5.0	26.0	(35)		
	Q9Y4L1	Hypoxia up regulated protein 1	111266	5.0	38.8	11(30)		
	P35579	Myosin 9	226390	5.3	32.6	22(83)		
**29**	P12109	Collagen alpha 1 VI chain	108461	5.1	53.9	21(120)	1.5↓	0.093
**30**	P12109	Collagen alpha 1 VI chain	108461	5.1	51.0	21(114)	2.0↓	0.007
**32**	Q12905	Interleukin enhancer binding factor 2	43035	5.0	38.7	8(11)	1.5↓	0.006
**34**	P08133	Annexin A6	75825	5.3	81.9	30(131)	1.8↑	0.037
	P08133-2	Isoform 2 of Annexin A6	72378	5.3	78.0	29(128)		
	P11142	Heat shock cognate 71 kDa protein	70854	5.2	64.6	10(64)		
**35**	Q03252	Lamin B2	67647	5.1	62.5	(62)	1.8↑	0.065
	P08107	Heat shock 70 kDa protein 1A 1B	70009	5.3	52.1	(42)		
	P08133-2	Isoform 2 of Annexin A6	72378	5.3	45.4	13(28)		
**36**	P02679	Fibrinogen gamma chain	51478	5.2	60.9	(69)	1.7↓	0.050
**37**	P02679-2	Isoform Gamma A of Fibrinogen gamma chain	49464	5.6	69.1	(55)	1.8↑	0.169
	P55072	Transitional endoplasmic reticulum ATPase	89265	5.0	81.9	(133)		
**38**	P02679-2	Isoform Gamma A of Fibrinogen gamma chain	49464	5.6	73.7	(112)	1.5↓	0.205
	O43707	Alpha actinin 4	104788	5.1	61.0	(65)		
	P55072	Transitional endoplasmic reticulum ATPase	89265	5.0	42.9	(27)		
**39**	P02679-2	Isoform Gamma A of Fibrinogen gamma chain	49464	5.6	70.0	(108)	1.7↓	0.013
	O43707	Alpha actinin 4	104788	5.1	68.7	(80)		
	P55072	Transitional endoplasmic reticulum ATPase	89265	5.0	36.6	(28)		

	P01023	Alpha 2 macroglobulin	163187	6.0	22.9	(32)		
	P35609	Alpha actinin 2	103788	5.2	19.2	(15)		
**40**	P02679-2	Isoform Gamma A of Fibrinogen gamma chain	49464	5.6	60.9	(122)	1.5↓	0.065
	O43707	Alpha actinin 4	104788	5.1	61.5	(66)		
	P01023	Alpha 2 macroglobulin	163187	6.0	34.9	(36)		
	P12814-2	Isoform 2 of Alpha actinin 1	102644	5.2	33.5	(29)		
	P55072	Transitional endoplasmic reticulum ATPase	89265	5.0	57.1	(37)		
	P35609	Alpha actinin 2	103788	5.2	25.7	(19)		
	Q08043	Alpha actinin 3	103176	5.2	21.2	(19)		
**41**	P02679-2	Isoform Gamma A of Fibrinogen gamma chain	49464	5.6	60.2	(108)	1.5↓	0.037
	P55072	Transitional endoplasmic reticulum ATPase	89265	5.0	44.9	(40)		
	P01023	Alpha 2 macroglobulin	163187	6.0	25.1	(31)		
**42**	P02768	Serum albumin	69321	5.9	29.6	7(53)	12.5↑	0.183
**43**	P07195	L lactate dehydrogenase B chain	36615	5.6	62.3	12(75)	3.3↑	0.178
**44**	P30040	Endoplasmic reticulum resident protein 29	28975	7.3	56.7	9(44)	2.0↑	0.165
**45**	P49189	4 trimethylaminobutyraldehyde dehydrogenase	53767	5.6	39.7	8(30)	2.3↓	0.021
**47**	P02768	Serum albumin	69321	5.9	86.7	(260)	3.5↓	0.000
**48**	P06396-2	Isoform 2 of Gelsolin	80590	5.5	60.9	20(84)	1.6↓	0.394
**49**	P30041	Peroxiredoxin 6	25019	6.0	83.0	10(61)	2.4↑	0.119
**50**	P02675	Fibrinogen beta chain	55892	8.3	51.3	11(92)	2.4↑	0.218
	Q15257-2	Isoform 1 of Serine threonine protein phosphatase 2A activator	36751	5.9	49.8	11(24)		
	P37837	Transaldolase	37516	6.4	38.3	9(19)		
**51**	Q9GZM7	Tubulointerstitial nephritis antigen-like	52352	6.5	40.3	6(43)	1.6↓	0.004
**52**	P23526	Adenosylhomocysteinase	47685	5.9	45.4	14(70)	2.4↑	0.010
**53**	P02675	Fibrinogen beta chain	55892	8.3	30.3	6(15)	3.0↑	0.065
**54**	P12110	Collagen alpha 2 VI chain	108511	5.8	28.9	(65)	2.6↑	0.003
**55**	P12110	Collagen alpha 2 VI chain	108511	5.8	34.2	(70)	2.6↑	0.155
**56**	P50395	Rab GDP dissociation inhibitor beta	50630	6.1	83.1	6(76)	1.5↑	0.235
	P02675	Fibrinogen beta chain	55892	8.3	65.2	14(78)		

**57**	P02675	Fibrinogen beta chain	55892	8.3	71.1	14(106)	2.0↓	0.101
**58**	P61163	Alpha centractin	42586	6.2	72.3	10(57)	2.7↑	0.005
	P27361	Mitogen activated protein kinase 3	43108	6.3	55.1	9(35)		
**59**	P02675	Fibrinogen beta chain	55892	8.3	78.8	14(92)	1.6↓	0.029
	Q9NVA2	Septin 11	49367	6.4	32.9	4(18)		
**61**	P06396-2	Isoform 2 of Gelsolin	80590	5.5	50.3	18(82)	3.2↑	0.001
	P02786	Transferrin receptor protein 1	84818	6.2	38.4	22(48)		
**62**	P02787	Serotransferrin	77013	6.8	60.0	17(171)	2.2↓	0.016
